# Non-Additive Transcriptomic Responses to Inoculation with Rhizobia in a Young Allopolyploid Compared with Its Diploid Progenitors

**DOI:** 10.3390/genes8120357

**Published:** 2017-11-30

**Authors:** Adrian F. Powell, Jeff J. Doyle

**Affiliations:** 1 Section of Plant Biology, School of Integrated Plant Sciences, Cornell University, Ithaca, NY 14853, USA; afp43@cornell.edu; 2 Boyce Thompson Institute, Ithaca, NY 14853, USA; 3 Section of Plant Breeding and Genetics, School of Integrated Plant Sciences, Cornell University, Ithaca, NY 14853, USA

**Keywords:** allopolyploidy, *Glycine*, nodulation, rhizobia, transcriptome

## Abstract

Root nodule symbioses (nodulation) and whole genome duplication (WGD, polyploidy) are both important phenomena in the legume family (Leguminosae). Recently, it has been proposed that polyploidy may have played a critical role in the origin or refinement of nodulation. However, while nodulation and polyploidy have been studied independently, there have been no direct studies of mechanisms affecting the interactions between these phenomena in symbiotic, nodule-forming species. Here, we examined the transcriptome-level responses to inoculation in the young allopolyploid *Glycine dolichocarpa* (T2) and its diploid progenitor species to identify underlying processes leading to the enhanced nodulation responses previously identified in T2. We assessed the differential expression of genes and, using weighted gene co-expression network analysis (WGCNA), identified modules associated with nodulation and compared their expression between species. These transcriptomic analyses revealed patterns of non-additive expression in T2, with evidence of transcriptional responses to inoculation that were distinct from one or both progenitors. These differential responses elucidate mechanisms underlying the nodulation-related differences observed between T2 and the diploid progenitors. Our results indicate that T2 has reduced stress-related transcription, coupled with enhanced transcription of modules and genes implicated in hormonal signaling, both of which are important for nodulation.

## 1. Introduction

Nodulation and whole genome duplication (WGD, polyploidy) are both notable phenomena in the evolutionary history of legumes (Leguminosae). Much progress has been made in recent years in understanding these independent phenomena; however, the potential interactions between the two remain poorly understood, which is particularly true with respect to the metabolic, genetic and expression-level changes that can alter nodulation responses in polyploids. 

The nitrogen-fixing root nodules formed in such symbioses are a key source of nitrogen for many legumes and account for important contributions to agricultural and natural systems [1,2]. Nodulation symbioses occur in a wide taxonomic diversity of legumes, and involve even more phylogenetically diverse bacteria (termed ‘rhizobia’) [3]. Many of the signaling components and molecular mechanisms required for the establishment of rhizobial symbioses have been elucidated (e.g., [4]). The signaling between partners involves Nod factors—lipochitooligosaccharides synthesized by rhizobia in response to Nod factor-inducing compounds (often flavonoids) from a host plant’s root—which are then perceived via plant receptors; variation in these several signaling components is believed to contribute to the diversity and specificity of interactions [5]. Next-generation sequencing approaches have also provided insights directly into the transcriptome-level responses of legumes to inoculation with rhizobia in model taxa. Libault et al. [6] compared responses in inoculated root hairs over time, Hayashi et al. [7] examined early responses to inoculation, and Barros de Carvalho et al. [8] studied whole soybean roots at later time points. Recently, laser capture microdissection has also been coupled with transcriptomic analysis to examine expression in specific tissue regions (e.g., [9,10]).

There have been no direct studies of the metabolic or expression-level interactions between polyploidy and processes of nodulation. However, ancient WGD has been suggested to have had a role in the evolution and refinement of nodulation in legumes [11,12,13]. With the publication of the *Medicago truncatula* genome, evidence of a WGD shared by many papilionoid legumes was coupled with evidence of duplicate gene retention and expression patterns to suggest that key genes involved in nodulation signaling evolved from genes duplicated by this WGD. The authors stated that “the WGD early in papilionoid evolution allowed the emergence of critical components in Nod factor signaling and contributed to the complexity of rhizobial nodulation observed in this clade” [11]. Polyploidy is known to cause changes at the level of gene expression that can impact phenotypes, including responses to abiotic and biotic factors [14,15,16], so it is plausible that direct effects on nodulation responses should also be observed. Patterns of non-additive expression at the whole transcriptome and individual gene levels in allopolyploids have been observed in numerous cases (reviewed in [17]), suggesting that substantial expression-level variability can occur. Expression of multiple parental homoeologues can be a source of variation, as expression of differing copies of enzymes, receptors or transcription factors, for example, and the degree to which homoeologous copies are expressed can lead to alterations in biological function. Thus, parental legacies and homoeologue expression biases can lead to numerous potential expression-level outcomes that, in turn, alter plant traits (e.g., [17,18,19,20]).

While transcriptomic studies have been used effectively to study nodulation and polyploidy as distinct phenomena, there have yet to be studies examining the interactions of the two phenomena in the same system. Here we examine the transcriptional responses of a recent allopolyploid to rhizobial inoculation and compare them to those of its diploid progenitors. Thus, we are inquiring into the effects of recent allopolyploidy on nodulation responses at the level of gene expression. We also examine the implications of homoeologue usage and parental legacies to expression-level responses to nodulation.

The *Glycine* subgenus *Glycine* allopolyploid complex presents an opportunity to study the intersection of nodulation and the effects of recent allopolyploidy. The complex consists of approximately 20 species, including several diploid species that have formed allopolyploids in various combinations within the last several hundred thousand years [21,22]. The capacity for nodulation in a number of species in this complex has been documented [23,24,25], and recent work has examined rhizobial interaction in relation to allopolyploidy in the system [26]. Transcriptomic approaches have been used in the complex to examine responses to light stress, as well as effects of polyploidy on photosynthesis, transcriptome size, and the translatome [27,28,29,30,31]. 

We used RNA sequencing (RNA-seq) to examine transcriptional responses to inoculation in the allopolyploid *Glycine dolichocarpa* (referred to throughout as T2), and its diploid progenitors *Glycine tomentella* (D3) and *Glycine syndetika* (D4). We were interested in determining the transcriptional patterns of the allopolyploid when compared to the progenitors, both transcriptome-wide and when considering specific sets of nodulation-related genes. Previous work found that T2 appeared to be broadly more responsive in early symbiotic signaling when inoculated with rhizobia than D3 and D4, had a greater propensity to form more nodules than either diploid, and tended to form a greater nodule mass than the D3 progenitor [26]. Thus, we expected that a transcriptomic analysis would provide insights into mechanisms at the whole transcriptome and individual gene levels that contribute to these responses to rhizobia in T2. Furthermore, in considering the regulation of expression levels in T2, we were interested in patterns of parental dominance, additivity and transgressive variation and how these relate to the response to rhizobial inoculation. Lastly, transcriptomic data were used to assess relative homoeologue expression within the allopolyploid and, thus, examine how the D3 and D4 homoeologues of key nodulation-related genes are deployed in response to inoculation. Through such analyses, we were able to identify transcriptional responses implicated in interactions with rhizobia that set the allopolyploid apart from one or both of its diploid progenitors.

## 2. Materials and Methods 

### 2.1. Plant Material

Three accessions for each of the three species were used to generate transcriptomes: G1364, G1403, and G1820 for *G. tomentella* (D3); G1300, G2073, and G2321 for *G. syndetika* (D4); G1134, G1188, and G1393 for *G. dolichocarpa* (T2). Seeds were surface-sterilized, nicked and placed in petri plates for germination under sterile conditions according to the protocol outlined in Powell and Doyle [26]. Seedlings were germinated for seven days before being transferred, in a laminar flow hood, to plates of solid Jensen’s nitrogen-free media [32,33]. Following the transfer to media, the seedlings were either mock-inoculated with sterilized water (also referred to as ‘control’ samples) or inoculated with a liquid culture of the rhizobial strain NGR234. Lyophilized NGR234 was obtained from the USDA-ARS Rhizobium Germplasm Resource Collection and revived according to provided protocols. The liquid culture was grown at 30 °C for five days in arabinose-gluconate (AG) media [32]. Prior to inoculation, the bacterial culture was pelleted and washed three times before being resuspended in sterilized water. The culture used to inoculate the seedlings had a concentration of approximately 10^7^ bacterial cells per mL as determined with a Helber counting chamber [32]. Each seedling was inoculated with 1 mL of bacterial culture. Seedlings in media plates were then placed in a growth chamber with a day:night cycle of 16h:8h, with temperatures of 22 °C during the day and 18.5 °C at night. Relative humidity was set at 60% and the light was 150 µmol·m^−2^·s^−1^. Similar growth conditions have been used previously in this system [27,31] and, in previous work with rhizobia [26], similar growth conditions on nitrogen-free media yielded approximately 95% nodulation of T2 plants by NGR234, indicating that such conditions can be conducive to efficient nodulation between specific symbiotic partners. After seven days, plant whole root tissue was harvested, frozen in liquid nitrogen and stored at −80 °C.

### 2.2. RNA Sequencing

Total RNA was extracted from pooled root tissue from multiple individuals per accession, and RNA-seq libraries were constructed for each accession using the pooled tissue. For each of the three accessions per species, two libraries were constructed: one from the control, mock-inoculated tissue and another from the tissue inoculated with NGR234. In this design, three samples were obtained under each treatment for each species. Total RNA was extracted using the AllPrep DNA/RNA/miRNA Universal Kit (QIAGEN, Hilden, Germany) with on-column DNase treatment (QIAGEN, Valencia, CA, USA). Single-end libraries were constructed and multiplexed using the TruSeq Stranded mRNA LT Sample Prep Kit (Illumina, San Diego, CA, USA).

The 18 libraries in this experiment were 6-plex sequenced. Sequencing was conducted using the Illumina HiSeq 2500 platform at the Cornell University Biotechnology Resource Center’s sequencing facility. Sequencing data are available through NCBI’s Sequence Read Archive (SRR5602634–SRR5602651).

### 2.3. RNA-Seq Data Processing

Fastq-mcf [34] was used to process reads; sequence reads were trimmed (with a minimum quality cutoff of 30) and short reads were removed (with a minimum read length of 50). Cleaned reads were mapped to the *Glycine max* genome (2.75_Wm82.a2.v1) using TopHat2 [35]. Uniquely mapped reads were obtained using the command samtools view -h input.bam|grep -w “@SQ\|@PG\|NH:i:1” and the htseq-count command [36] was used to count reads mapped to each exon.

### 2.4. Homoeologue Mapping

Homoeologues were identified and mapped using the approach developed in Bombarely, Coate and Doyle [21]. In order to separate reads in T2 samples based on diploid progenitor origin, we generated reference transcriptomes for the D3 and D4 progenitors using the RNA-seq root samples described above, along with additional sequence data from paired-end DNA-seq samples that were also generated from root tissue, using Samtools and Mpileup [37], Gffread [38], and Bowtie2 [39] to generate a reference transcriptome. Reads from T2 samples were then mapped to a concatenated reference comprising both the D3 and D4 reference transcriptomes using HISAT2 [40]. Reads were then separated based on preferential mapping to each of the diploid reference transcriptomes using the SeparateHomeolog2Sam. The separated homoeologue-specific reads were then mapped back to the reference soybean genome and reads were counted using htseq-count [36]. For estimates of homoeologue usage percentages for the overall transcriptome, genes with zero counts for both homoeologous copies were excluded from the calculation for each accession; of the 56,044 genes in the transcriptome, following homoeologue separation and mapping, an average of 37,599 were used for mock-inoculated samples and an average of 36,313 were used for inoculated samples.

### 2.5. Expression-Level Analyses

The R package DESeq2 [41] was used to identify differentially expressed genes (DEGs) between paired control and inoculated samples within species, using a false discovery rate (FDR) of 0.05 to control for multiple comparisons. Principal component analysis was conducted using read counts transformed using the rlog() function in DESeq2. The approach to analysis of expression-level dominance was adapted from Yoo, Szadkowski and Wendel [18]. Expression levels were analyzed separately in mock-inoculated samples and inoculated samples using the edgeR package [42], comparing across species. Accession samples for each species were first normalized, dispersions were estimated and differential expression was assessed between the allopolyploid T2 and each diploid progenitor using exactTest() [18,43]. Analysis was restricted to genes that had at least one read per million in at least three samples (accessions) in each comparison [43]. Differential expression between species was determined with a FDR of 0.05 using the Benjamini–Hochberg adjustment [44]. Genes with differential expression in the allopolyploid were then placed into one of twelve possible categories of differential expression patterns as defined by Rapp et al. [45] and Yoo, Szadkowski and Wendel [18].

For the analysis of known nodulation-related genes, a list of such genes was obtained from the Schmutz et al. [46] soybean genome paper. The corresponding gene IDs in the 2.75_Wm82.a2.v1 soybean genome were obtained and this resulted in a set of 47 genes used for ordination and fold-change analysis, including genes encoding symbiotic signaling receptors, transcription factors and nodulins. For the expression-level analysis based on the approach of Yoo, Szadkowski and Wendel [18] described above, several genes did not meet the cutoff for inclusion in the analysis; as such, 32 genes were used in comparisons between control samples and 33 genes were used in comparisons between inoculated samples.

### 2.6. Weighted Gene Co-Expression Network Analysis (WGCNA)

Expression networks were constructed, and co-expression modules were identified, using the R package WGCNA [47,48] and count data normalized using the rlog() function of the DESeq2 package [41]. Applying the approximate scale-free topology criterion [47,49], we selected a threshold power of β = 5. A maximum block size of 20,000 and a minimum module size of 100 were specified in the blockwiseModules() function. We conducted the signed network construction using all inoculated and mock-inoculated samples, grouping genes by average linkage hierarchical clustering; determination of modules was achieved with a dynamic tree cutting algorithm. We examined module eigengene expression in the inoculated samples in relation to trait data obtained from Powell and Doyle [26] for the same accessions as those used in the present study, including root hair deformation ratio, total nodule mass per plant, total number of nodules per plant, and percent of plants with nodules per accession; the module eigengene is defined as the first principal component for a module [47]. Module eigengenes are summaries that give the expression value of an average, representative gene for modules, and these values can be compared among samples. We also identified hub genes based on correlations between gene significance for the root hair trait and module membership.

## 3. Results and Discussion

### 3.1. T2 Exhibited Overall Expression-Level Intermediacy, but Had Fewer Differentially Expressed Genes Compared to Diploids 

**Figure 1:**
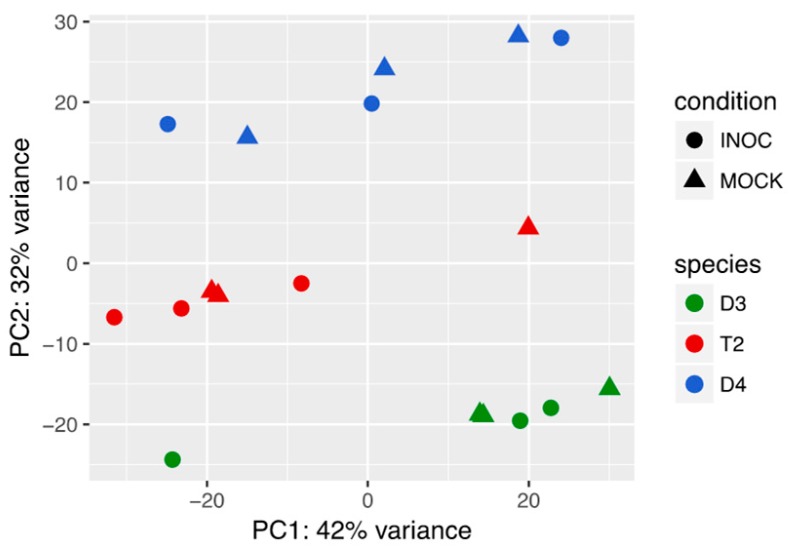
Principal component (PC) analysis of all D3 (*Glycine tomentella*), D4 (*Glycine syndetika*), and T2 (*Glycine dolichocarpa*) samples, conducted using rlog transformed expression values for the top 500 heterogeneously expressed genes across all samples. ‘INOC’ indicates samples inoculated with rhizobia; ‘MOCK’ indicates control samples that were mock-inoculated.

The T2 transcriptomes tended towards overall intermediacy in expression levels (Figure 1). Principal component analysis of the transcriptional profiles showed the samples mainly grouping by species. The first two principal components accounted for approximately 74% of the variation between samples. When the analysis was restricted to a set of known nodulation-related genes compiled by Schmutz et al. [45], T2 samples appeared again to be intermediate to the diploids (Figure S1).

**Figure 2:**
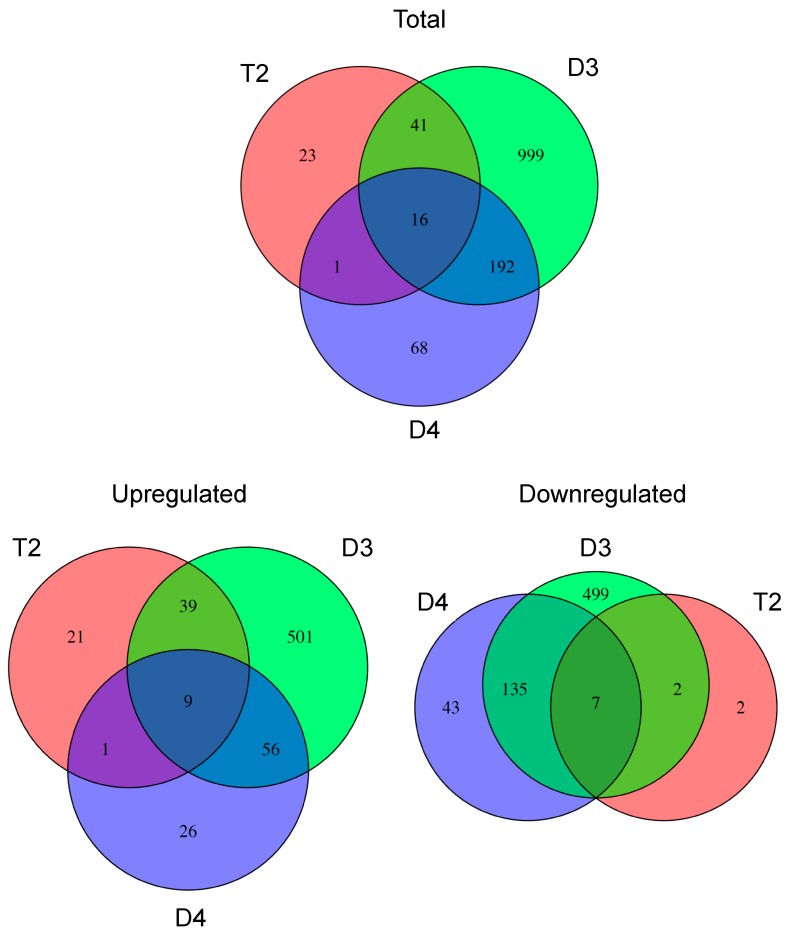
Venn diagram showing overlap between D3, T2, and D4 for all genes differentially expressed in response to inoculation in each species, as well as for sets of upregulated and downregulated genes considered separately.

**Figure 3:**
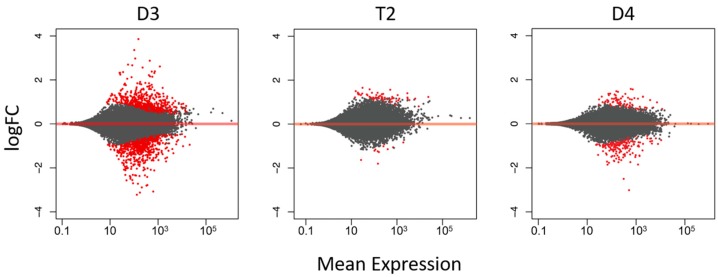
Pairwise comparisons of differential gene expression, showing average log fold-change (FC) in expression between inoculated and control samples relative to mean expression (mean of normalized counts of all samples) for species D3, D4 and T2. Red dots are genes that were significantly differentially expressed (false discovery rate (FDR) < 0.05), while gray dots indicate genes that were not significantly differentially expressed.

In addition to this broad expression-level intermediacy, T2 showed a lesser overall transcriptional response to inoculation compared to its diploid progenitors based on the number of genes that were significantly differentially regulated following exposure to rhizobia. In total (i.e., including both upregulated and downregulated genes), T2 had 81 significantly DEGs, while D4 and D3 had 277 and 1248 significantly DEGs, respectively (Figure 2). The greater responses in D3 and D4 compared to T2 were also illustrated in ‘MA’ plots, which show additional information on the magnitude of fold-changes of gene expression for individual genes, by plotting log ratios (M) of expression against mean average (A) expression for each gene (Figure 3). 

From the overall transcriptional responses, then, we can observe that, although the allopolyploid T2 samples were generally transcriptionally intermediate to D3 and D4 when considering the most variably expressed genes, T2 had a reduced transcriptional response to inoculation with rhizobia when compared to either of its diploid progenitors. A similar reduced response was observed in T2 in the context of light stress [27], where the allopolyploid was not stressed to the same degree as the diploids when challenged with elevated light levels and had reduced transcriptional responses for stress-related genes [27]. We therefore sought to explore further whether a similar phenomenon might account for transcriptional differences between T2 and its diploid progenitor D3.

### 3.2. Hydrogen Peroxide and Oxidative Stress Responses were Differentially Regulated in D3

**Figure 4:**
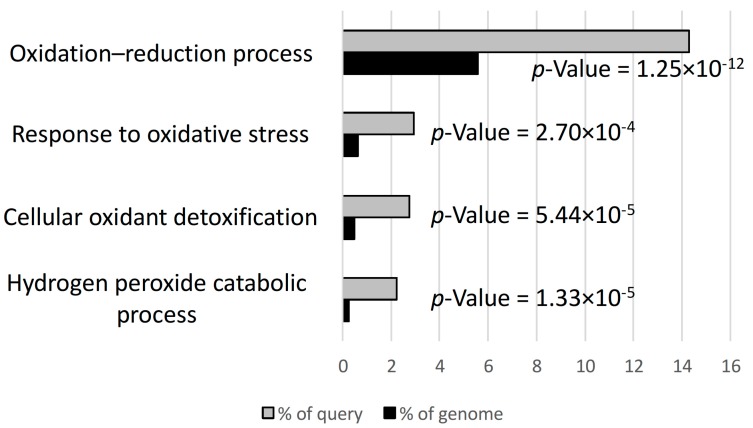
The four most highly significant gene ontology (GO) biological process parent terms resulting from a GO overrepresentation analysis of the D3 genes upregulated by inoculation treatment. Overrepresentation is shown by comparing genes in a given term as a percentage of the genome vs. as a percentage of the set of D3 upregulated genes.

To explore further the nature of the enhanced transcriptional response to inoculation in D3, we examined the gene ontology (GO) terms that were significantly overrepresented among upregulated and downregulated genes. Not surprisingly given the disparity in DEG numbers, in both classes, D3 had more GO terms with overrepresented genes than either T2 or D4. In the sets of T2 upregulated and downregulated DEGs, no terms of molecular function, biological process or cellular enrichment were significantly overrepresented. D3, however, had several significantly overrepresented terms related to oxidative stress among upregulated genes (Figure 4), including the biological process term related to hydrogen peroxide catabolism. These GO terms are suggestive of an enhanced stress and defense response in D3 at the level of the transcriptome; this was also explored in terms of fold changes for individual genes. Libault et al. [6], in their study of the *G. max* root hair transcriptomes inoculated with rhizobia, highlighted a set 11 of defense-related, nodulation-responsive genes. We used this set to query the list of genes regulated by inoculation treatment across all samples of D3, T2 and D4. Three of the 11 defense-related genes were found in our set of nodulation-responsive genes; of these, two genes encoding peroxidases were identified, Glyma.02G259300 and Glyma.14G053600, and in both cases D3 showed the greatest upregulation, D4 was the progenitor upregulating them the least, and T2 was either the same as D4 or intermediate (Figure S2). One factor contributing to T2’s lesser overall transcriptional response, then, was that it had an apparently reduced upregulation of stress and defense response transcripts when compared to D3. However, this does not provide insight into T2’s decreased response compared to D4.

The production of reactive oxygen species (ROS), including hydrogen peroxide, contributes to the hypersensitive response and is a well-characterized aspect of responses to bacterial pathogens in plants, including *G. max* [50,51]. The result obtained here was also consistent with 3,3′-diaminobenzidine (DAB) staining assays, where D3 showed greater hydrogen peroxide evolution when exposed to a strain of *Ensifer fredii* rhizobia than did T2 or D4 [52]. Indeed, legumes show a variety of defense responses following inoculation with rhizobia, though these tend to be rapidly suppressed in the case of effective symbionts. At the transcriptional level, expression of defense-related genes is upregulated in *G. max*, *Lotus japonicus*, and *M. truncatula* in the early stages of response to inoculation [6,53,54]. The evolution of reactive oxygen species (ROS; specifically, hydrogen peroxide) has also been observed upon inoculation. The rate of production of ROS decreases following application of compatible Nod factors [55], and inoculation with mutant rhizobia that are defective in Nod factor synthesis leads to increased production of ROS in *Medicago sativa* [56]. The initial burst of hydrogen peroxide is also potentially necessary to trigger certain nodulation-related processes and control gene expression that is conducive to nodulation [57,58]. This initial burst, however, appears to occur at an earlier time point than that which was sampled here; at this later time point, one would expect defense responses to have attenuated. Further, there is some evidence that alterations to defense-related mechanisms and increased responses can also lead to reductions in the size of individual nodules and total nodule mass per plant (e.g., [59,60]). The apparent transcriptional upregulation of defense-related genes in D3 may therefore, in part, account for the reduced nodule mass per plant previously observed in D3 relative to T2 when inoculated with the same strain of rhizobia [26]. D3, by maintaining a stronger defense response, could be inhibiting progression of infection and symbiosis.

### 3.3. Implications of Expression-Level Dominance for Nodulation

In addition to overall transcriptome-level responses to inoculation, we examined regulation of genes known to be involved in nodulation. Nine of the 47 nodulation genes from Schmutz et al. [46], whose list included genes implicated at different stages of symbiosis, were among those regulated by inoculation treatment alone across all samples (FDR < 0.05). These genes had a variety of fold-change patterns in specific transcript accumulation between control and inoculated samples for each species (Figure S3). For four of these genes, T2 showed intermediate fold-change patterns between D3 and D4, with D3 showing the greatest fold change in each case. It is noteworthy that the *Nodule Inception* gene (*NIN*) was among these genes, since it is a key transcriptional activator in downstream nodulation responses [61]. If T2 is able to upregulate *NIN* to a greater degree than one of its progenitors, it could increase the degree of symbiotic response over that progenitor.

How transcriptional responses of allopolyploids compare with those of their progenitors is an issue of considerable biological importance. One question relates to expression levels in the allopolyploids compared to those of the diploid progenitors, asking whether the sum of the two homoeologous copies in the polyploid is similar to that of one diploid progenitor or the other (expression-level dominance: [18,62]), versus being expressed at mid-parent or transgressive levels. Furthermore, if there is novel gene expression or silencing relative to the diploid progenitors, this could alter biotic interactions. Here, the meaning of ‘silencing’ and ‘novel expression’ were adopted from Yoo et al. [18] and were evaluated in a similar manner, where silencing meant an absence of expression in all the allopolyploid accessions of genes expressed in one or both diploids and novel expression meant expression of genes in at least one allopolyploid accession that were not expressed in either diploid. 

In the a priori set of nodulation genes, there was no evidence for cases of silencing or novel expression in the allopolyploid. Studies using synthetic and natural hybrids and allopolyploids have demonstrated that effects on polyploids are produced by both genome merger and genome doubling, as well as by evolutionary divergence over time. While expression levels in an allopolyploid can be a result of the parental legacy of expression patterns in progenitors or can relate to novel changes due to allopolyploidization [19], there is evidence that the genome merger itself alters regulatory interactions and leads to substantial changes in expression levels, which are further enhanced over evolutionary time [17]. As the time since the genome merger increases, expression-level dominance, as well as the number of transgressively expressed genes, also increases; this has been shown in the case of cotton [18]. The absence of silencing observed in the nodulation genes studied here is understandable given the relatively recent formation of T2 (within the last several hundred thousand years [21,22]), and a similar general absence of silencing has previously been observed in T2 related to expression of genes involved in photosynthesis and photoprotection [27,29]. 

Most of the genes in the nodulation set did not show interspecific expression differences (Table 1, “No change”). Using the expression categories and approach for testing significant differential expression of genes between species developed by Yoo et al. [18] for allopolyploid cotton, we classified the 10 nodulation genes that showed differential expression between species in mock-inoculated samples of T2, and the eight genes differentially expressed in the samples inoculated with rhizobia (Table 1 and Table S1). Under both treatment conditions, for nodulation genes showing patterns of expression-level dominance, the allopolyploid tended to have more genes expressed at the level of the diploid progenitor that had higher expression (e.g., comparing the sum of categories II and IV with the sum of XI and IX yields 4 and 2, respectively, for control samples, and 2 and 1, respectively, for inoculated samples). 

Despite the general intermediacy of T2 in overall transcriptional analyses, then, certain key nodulation genes examined here showed expression-level dominance, where the allopolyploid T2 had similar expression to that of the higher-expressing diploid progenitor, and transgressive upregulation in T2 when compared to D3 and D4. Transgressive upregulation was observed for a nodulin gene (*NODULIN-33* in both control and inoculated samples) and an early nodulin gene (*ENOD8* in inoculated samples), showing an enhanced capacity for expression-level responses to inoculation in T2. The *Nodulating signaling pathway 2* gene (*NSP2*) was also transgressively upregulated in inoculated T2 samples, and *Nodule autoregulation receptor kinase* (*NARK*) was upregulated in control samples. NSP2 is a member of the GRAS family of transcription factors and is a key regulator forming a complex with NSP1 that binds to the promoters of several nodulation-induced genes essential for the symbiotic signaling response, including *NIN* and *Ethylene responsive factor required for nodulation 1* (*ERN1*) [63,64]. In terms of expression-level dominance, it is notable that in the control samples T2, along with D3, expressed the two Nod factor receptor genes *NOD factor receptor 1* (*NFR1*) and *NFR5* more highly than did D4, thus potentially enabling enhanced perception of rhizobial signaling in T2 relative to D4. T2 may be a better nodulator than its progenitors [26] because of its enhanced expression of nodulation genes.

In conjunction with its reduced overall transcriptional response, T2 appears to have a reduced stress and defense response relative to D3, while also expressing several key nodulation-related genes at or above the level observed in one or both diploid progenitor species. This result further highlights the importance of a balance in the interaction between nodulation and defense responses required for functional nodulation symbioses.

### 3.4. Implications of Homoeologue Usage for Nodulation

**Figure 5:**
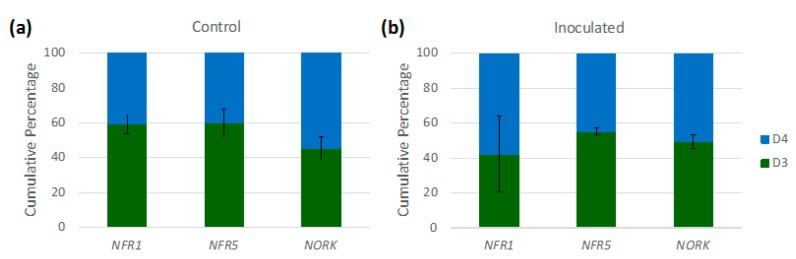
Homoeologue usage with respect to three key nodulation-related signaling receptors, in (**a**) control (mock-inoculated) and (**b**) inoculated samples of allopolyploid T2. Error bars indicate standard deviation.

The expression of multiple homoeologous symbiotic receptors has the potential to enhance nodulation interactions [65]. We found that allopolyploid T2 expressed multiple homoeologous copies of key symbiotic receptors (Figure 5), and in the examination of the percentages of D3 and D4 read mapping in T2 for these individual receptor genes, there were also no clear patterns of bias toward either progenitor (Table S2). The expression of both homoeologues of these receptors and the absence of a clear pattern of usage bias has implications for the symbiotic capacity of the allopolyploid. The Nod factor receptors NFR1 (Glyma.02g270800) and NFR5 (Glyma.11g063100) form heterodimers and, together, partly mediate specificity in interactions between legumes and rhizobia [5,66]; compatibility between the structure of the Nod factors and these Nod factor receptors can contribute to determining the symbiotic response. Thus, by expressing both homoeologous copies of these receptors, as we found in both mock-inoculated and inoculated samples, the allopolyploid T2 has the potential to increase its range of symbiotic partners. Similarly, NODULATION RECEPTOR KINASE (NORK) is an important receptor that constrains nodule formation; it appears to confer additional specificity between host and symbiont [4,67]. In allopolyploid T2, as with *NFR1* and *NFR5*, both D3 and D4 homoeologous copies of *GmNORK* (Glyma.09g202300) are expressed, thereby potentially enabling symbiotic interactions with a larger set of rhizobial genotypes. Although expression of multiple copies of such symbiotic receptors is not a sufficient condition for an enhanced symbiotic range, it does constitute a necessary condition; without it, such an increase in potential symbiont diversity mediated by receptor diversity would not be possible [65]. 

It should be noted that the three species studied here have homoeologues of these receptors from a previous whole genome duplication shared with *G. max* (*NFR1* homoeologue: Glyma.14g046200; *NFR5* homoeologue: Glyma.01g179100; *GmNORK* homoeologue: Glyma.01g020100). However, previous work in *G. max* has shown that, for *NFR1*, one copy (Glyma.02g270800) tends to be more highly expressed and is more relevant for nodulation [68]; these findings were consistent with those of the present study, where we found that *NFR1* homoeologue Glyma.14g046200 and *NFR5* homoeologue Glyma.01g179100 were expressed at negligible levels, while Glyma.02g270800 and Glyma.11g063100 were relatively highly expressed. Similarly, the *GmNORK* homoeologue Glyma.09g202300 was found to be primarily responsible for symbiotic functions [69]. Thus, in the results presented here on homoeologue expression in T2, we have focused on the receptors believed to be primarily involved in mediating symbiotic responses, and the results presented relate only to these genes.

We also explored homoeologue usage more generally in response to inoculation; if a shift in homoeologue usage following inoculation were detected, this could signal preferential mobilization of one subgenome in the process of nodulation. However, T2 shows essentially equal usage of its D3 and D4 homoeologues and paired *t*-tests detected no significant effects of inoculation on homoeologue usage, both transcriptome-wide and for the set of nodulation genes (Figure S4).

### 3.5. Non-Additive Expression Patterns of Nodulation-Related Modules and Hub Genes Identified by Network Analysis

To complement our examination of transcriptional responses using a set of known, pre-defined nodulation-related candidate genes, we sought to examine expression patterns of co-expressed gene modules and hub genes that were not defined a priori but, rather, were identified as relevant to nodulation through analysis of the transcriptome data from D3, D4, and T2. Weighted gene co-expression network analysis (WGCNA) has been used to explore gene expression in polyploids (e.g., [70,71]); here, we used WGCNA with a primary focus on understanding key nodulation-related processes and how these varied among the three species. First, we identified co-expression modules across all species; the analysis identified 46 modules of co-expressed genes, each assigned an identifying color label by WGCNA (Figure S5). Collectively, these modules contained 45,697 of the 56,044 genes represented in the transcriptomes (10,347 were excluded from the analysis for having too many missing values or zero variance). The ‘grey’ module, reserved for genes that could not be placed into any co-expression module, contained 2791 genes.

**Figure 6:**
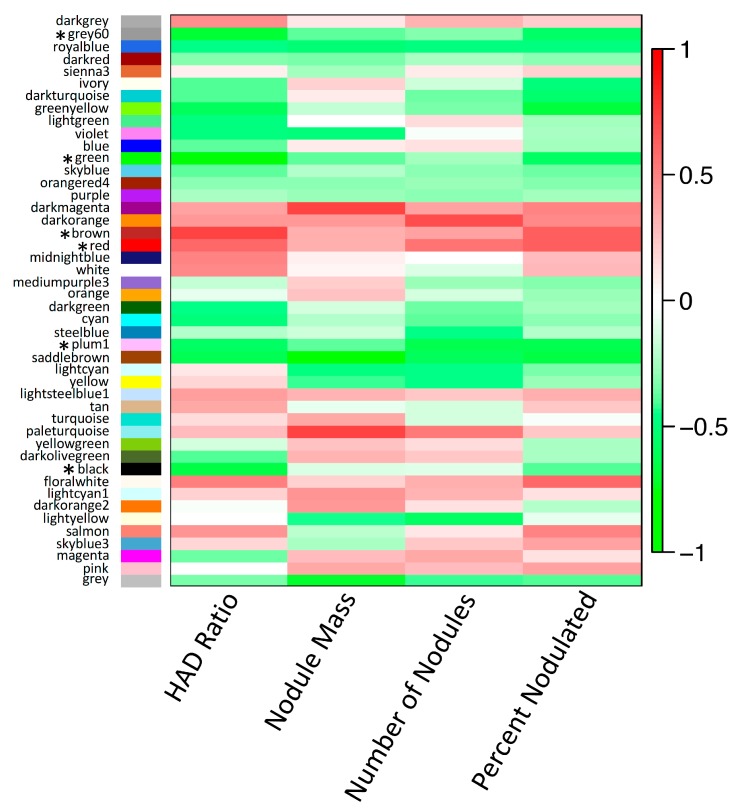
Correlation heatmap of eigengenes for all weighted gene co-expression network analysis (WGCNA) modules with nodulation-related trait data from [26]. Traits include the root hair deformation ratio (HAD Ratio), total nodule mass per plant (Nodule Mass), total number of nodules per plant (Number of Nodules), and percentage of plants nodulated per accession (Percent Nodulated). The heatmap is color-coded according to the Pearson correlation coefficient legend. Modules for which eigengenes are significantly correlated with HAD Ratio (*p* < 0.01) are indicated with asterisks.

We then assessed the correlation between the expression level of the eigengene summarizing each module and four traits in inoculated samples (Figure 6). For subsequent analyses, we focused on the root hair deformation trait (“HAD Ratio” in Figure 6), due to the transcriptome sampling corresponding most closely in time to the measurements of this trait, and its proximal relevance to early signaling and transcriptional responses in the nodulation symbiosis; however, while we expected correlated modules to be relevant to nodulation-related processes, we did not expect a direct mechanistic connection with the root hair deformation trait. The six modules with eigengene expression that showed significant correlations with root hair deformation (*p* < 0.01) formed a set of modules of interest implicated in nodulation-related processes (modules marked with asterisks in Figure 6).

**Figure 7:**
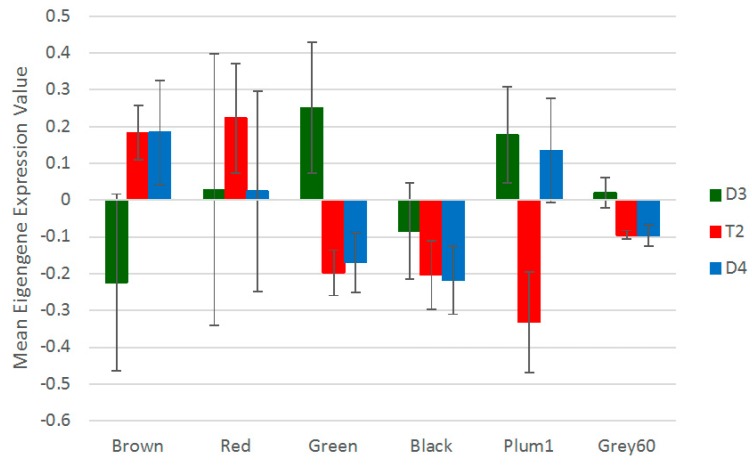
Expression of WGCNA module eigengenes, compared across species for each of the modules that were most highly correlated with the root hair deformation trait. Values shown are species means ± standard deviation.

Modules can be characterized and expression can be compared between species using module eigengene expression. In the module eigengene comparison between species for the six modules of interest identified by correlation with root hair deformation, there was significant differentiation in expression between species (i.e., “species” was a significant factor) for the ‘brown’ (*F*_2, 6_ = 5.957, *p* = 0.0376), ‘green’ (*F*_2, 6_ = 13.51, *p* = 0.006), ‘grey60’ (*F*_2, 6_ = 15.22, *p* = 0.00447) and ‘plum1’ (*F*_2, 6_ = 12.83, *p* = 0.00681) modules. In the case of the ‘brown’ module, whose eigengene expression was positively correlated with root hair deformation, D4 eigengene expression was higher than D3, with T2 expression at the higher level of D4 (Figure 7). With the ‘green’ and ‘grey60’ modules, the pattern was similar to the ‘brown’ module except that these modules were negatively correlated to the trait and D4 and T2 both expressed the eigengene at lower levels than D3 (Figure 7). For the ‘plum1’ module, which was negatively correlated with the root hair trait, a transgressive expression pattern was observed in T2, whose eigengene expression was reduced relative to D3 and D4 (Figure 7).

To understand more about the composition of the nodulation-associated modules for which species was a significant factor, GO overrepresentation tests with the ‘molecular function complete’ and ‘biological process complete’ annotation data sets were used to assess which types of transcripts were associated with modules to a greater degree than expected. Of the four modules associated with nodulation and also showing significant differences between species, the ‘brown’ module had the greatest number of most highly significant terms; ‘grey60’ had the significantly overrepresented biological process and molecular function terms of oxygen transport (*p* = 1.08 × 10^−2^) and oxygen transport activity (*p* = 8.59 × 10^−3^), while the other two modules had no overrepresented terms. The top four most significantly overrepresented parent terms for the ‘brown’ module are shown in Figure S6. These included regulation of ARF (ADP-ribosylation factor) protein signal transduction (biological process; *p* = 1.76 × 10^−4^), ARF guanyl-nucleotide exchange factor (ARF-GEF) activity (molecular function; *p* = 1.40 × 10^−4^), Golgi vesicle transport (biological process; *p* = 2.61 × 10^−3^), and intracellular protein transport (biological process; *p* = 4.61 × 10^−2^). The overrepresentation of ARF-GEF proteins is relevant because auxin accumulation and auxin-related signaling are believed to be critical for stimulating cell division during nodule organogenesis; ARF-GEF proteins are key regulators of the localization of auxin Pin formed (PIN) transporter proteins, and the localization of PIN proteins alters local auxin levels [72,73,74]. The expression of the ‘brown’ module eigengene in T2 at the level of the high-expression parent (D4) compared to D3 thus suggests a greater auxin-related signaling response to inoculation, which is implicated in the process of symbiotic development and nodule formation.

The second summary approach for assessing expression involves identifying hub genes for each module. Hub genes can be defined by setting criteria to identify genes in a given module that have a high correlation with root hair deformation (gene significance) and a high correlation with the module eigengene’s expression (module membership), and their expression patterns can be compared in the various samples. For the six modules identified as significantly related to the root hair trait, the genes of these modules generally showed module membership that was highly correlated with gene significance for the trait (Figure S7). Genes with both high module membership (high within-module connectivity) and high gene significance for the root hair trait were identified as hub genes of interest for the trait. With a criterion of gene significance greater than 0.7 and module membership greater than 0.7 as the minimum threshold, the ‘plum1’ and ‘grey60’ modules did not have any genes that were identified as hub genes and were thus excluded from this analysis. Three to five hub genes met the minimum threshold of gene significance and module membership for the ‘green’, ‘black’, and ‘red’ modules. The ‘brown’ module contained 24 hub genes (of which 23 had sufficient counts for subsequent testing and binning), even when a more stringent criterion (both gene significance and module membership greater than 0.8) was applied. 

The hub genes identified for each module were then classified according to the relative expression categories (Table S3) used for the known, a priori set of nodulation genes (as in Table 1). In the ‘red’ and ‘black’ modules, none of the hub genes showed significant differences in pairwise comparisons, as was the case for two of the hub genes from the ‘green’ module, while one hub gene in this module had a pattern of additivity. In the ‘brown’ module, a majority of the hub genes had a pattern of D4 expression-level dominance. This complements the eigengene expression results for this module. While the hub genes in this category did not show any obvious relationship to each other or to nodulation in terms of their identity, some of the genes are implicated in ethylene signaling: Glyma.03G251700 is annotated as a signal transduction histidine kinase ethylene sensor; Glyma.04G147000 is annotated as an F-box leucine rich repeat protein. The homologue of Glyma.04G147000 in *Arabidopsis thaliana* is *EBF1*, a negative regulator involved in the ethylene response pathway [75]. Ethylene is typically considered an inhibitor of nodulation, with ethylene-insensitivity leading to supernodulation phenotypes [76,77] and it is possible that ethylene signaling is involved in a Nod factor-triggered negative feedback mechanism [78], though the relationship between ethylene and nodulation appears to be complex (e.g., [79,80]). In connection with the GO analysis results for the ‘brown’ module discussed above, ethylene signaling has also been implicated in alterations to auxin transport during nodulation, and is coupled with changes in gene expression of the auxin efflux transporters *MtPIN1* and *MtPIN2* in *M. truncatula* [81]. Generally, given the evidence for a negative relationship between ethylene signaling and nodulation, if T2 expresses genes associated with negative regulation of ethylene signaling at greater levels than D3, this would also be associated with an enhanced capacity for nodule formation.

## 4. Conclusions

In this study, expression in allopolyploid T2 transcriptome samples was broadly intermediate relative to its diploid progenitors; however, our results also indicated that the allopolyploid had a reduced overall transcriptional response to rhizobia, in terms of genes regulated in response to inoculation, compared with one or both diploid progenitors, while having an enhanced symbiotic response. Reduction of transcriptional defense-related responses can play a role in modulating the response to rhizobia, enhancing the outcome of the interaction.

Furthermore, apparent expression-level intermediacy that is revealed, for example, by analyses of the most highly heterogeneously expressed genes can also mask other expression patterns underlying biotic responses. Here, non-additive patterns were observed in the analyses of pre-defined nodulation-related genes and modules identified through network analysis. When analyses were constrained to these sets of genes, patterns of expression-level dominance and transgressive patterns of expression (but not homoeologue usage biased consistently toward one progenitor) were observed in T2. These provide evidence of gene regulation on the part of the allopolyploid, in response to rhizobial inoculation, that is distinct from that of one or both diploid progenitors and deviates from expectations of additivity. The transgressive upregulation of certain nodulation-related genes and modules or their expression at levels of the higher-expressing diploid, coupled with a reduced defense response at the level of gene expression, provide a foundation for understanding the enhanced symbiotic responses in allopolyploid T2 observed when it is inoculated with the NGR234 strain of rhizobia [26]. Additional ongoing studies of plant signaling metabolites are revealing further differences between T2 and its diploid progenitors, which complement the results discussed here, particularly with respect to explaining the differences between T2 and D4, in terms of their propensities for forming nodules with strains of rhizobia other than NGR234 [82]. Thus, the present study is the first step towards a complete understanding of mechanisms by which allopolyploidy has affected nodulation in *Glycine* subgenus *Glycine*.

## Figures and Tables

**Table 1:**
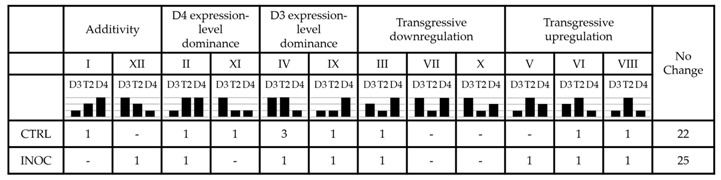
Expression patterns for nodulation-related genes from Schmutz et al. [45]. The twelve categories represent possible patterns of differential expression in the allopolyploid relative to its diploid progenitors, and have been adapted from [45]. For pairwise comparisons, control (‘CTRL’) and inoculated (‘INOC’) samples were analyzed separately.

## Supplementary Materials

The following are available online at www.mdpi.com/2073-4425/8/12/357/s1. Figure S1: PCA of all D3, D4, and T2 samples, generated using variance-stabilized expression values for a set of pre-defined, known nodulation genes. Samples are designated by the appropriate species prefix (D3, T2, D4); samples C1–C3 are control samples in each species and samples R1–R3 are the corresponding samples inoculated with rhizobia, Figure S2: D3 shows greater upregulation of three defense-related genes, including two peroxidase genes, in response to inoculation compared to T2 or D4. Single asterisks indicate expression differences between inoculated and control samples with an unadjusted *p*-value < 0.05. Double asterisks indicate expression differences with adjusted *p*-value < 0.05 (Benjamini–Hochberg adjustment), Figure S3: Log fold change (logFC) in expression of inoculation-responsive, known nodulation genes in inoculated relative to control samples within each species. Single asterisks indicate differences in expression between inoculated and control samples with an unadjusted *p*-value < 0.05. Double asterisks indicate differences in expression with a FDR < 0.05 (Benjamini–Hochberg adjustment), Figure S4: Homoeologue usage estimates for the overall transcriptome and for sets of nodulation-related genes, in control (mock-inoculated) and inoculated samples of allopolyploid T2 (*n* = 3). Stacked bars show mean percentages for each homoeologue. Error bars indicate standard deviation, Figure S5: Gene dendrograms obtained by WGCNA using blockwise network construction and all inoculated and mock inoculated transcriptome samples. The colors below the dendrograms show module assignment determined by the Dynamic Tree Cut. The blockwise method is used due to the size of the data set and computational constraints, and there is no particular significance to the attribution of a module to a given block, Figure S6: The four most highly significant GO biological process and molecular function parent terms resulting from a GO overrepresentation analysis of ‘brown’ module member genes. The figure illustrates this overrepresentation by showing the genes in each term as a percentage of the genome and as a percentage of the set of ‘brown’ module genes. For each term (B) indicates a biological process; (M) indicates a molecular function, Figure S7: Scatterplots of module membership (*x*-axis) relative to gene significance (*y*-axis) for all genes in each of the ‘brown’, ‘red’, ‘black’, ‘green’, ‘grey60’, and ‘plum1’ modules, which were the six modules that were significantly correlated with root hair deformation. Each circle in the plots represents a gene in the given module, Table S1: Identity and description of genes placed into the twelve categories of expression patterns shown in Table 1, Table S2: χ^2^ tests for deviations from 50:50 expression of homoeologues for genes encoding receptors involved in nodulation-related signal perception, Table S3: Expression patterns for hub genes from selected WGCNA modules.
