# Supplementary figures and images for: Non-Additive Transcriptomic Responses to Inoculation with Rhizobia in a Young Allopolyploid Compared with Its Diploid Progenitors
Source: Genes (Basel). 2017 Nov 30;8(12):357. doi: 10.3390/genes8120357 (PMC5748675; doi:10.3390/genes8120357)

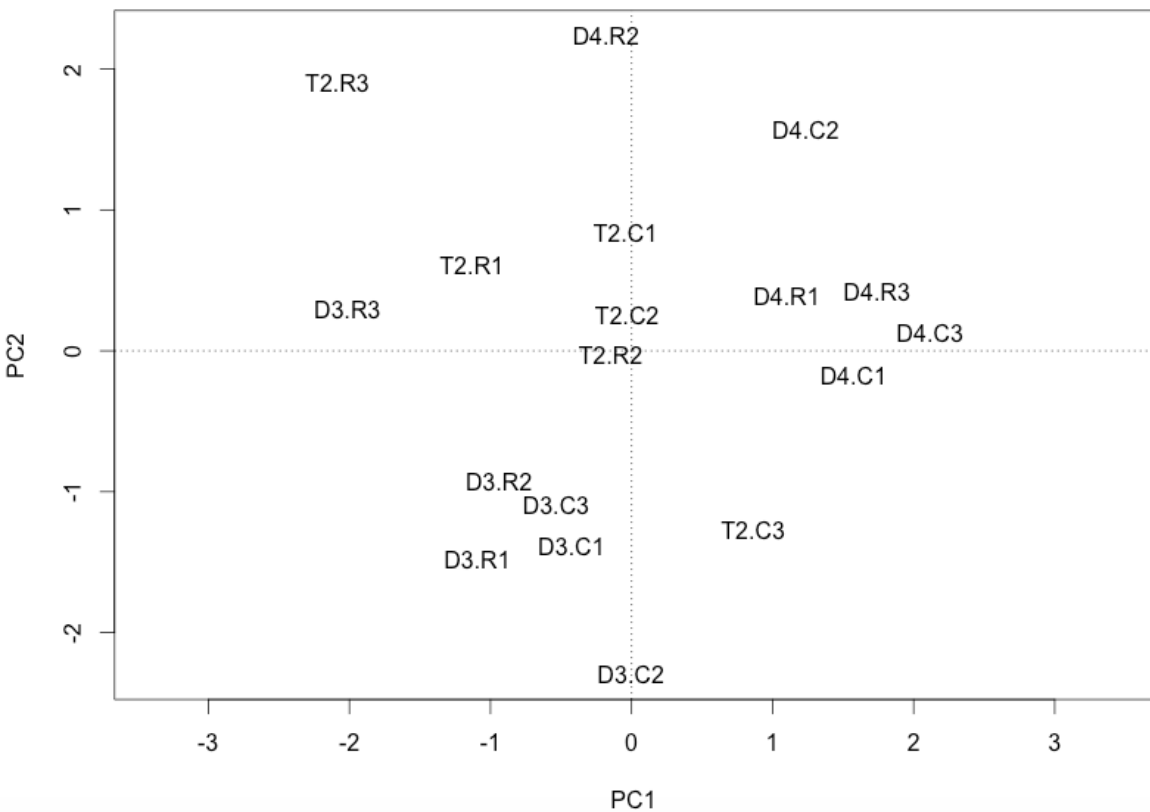

Supplement: Supplementary file 1 [file genes-08-00357-s001.zip › Supplemental_Fig_S1.pdf]

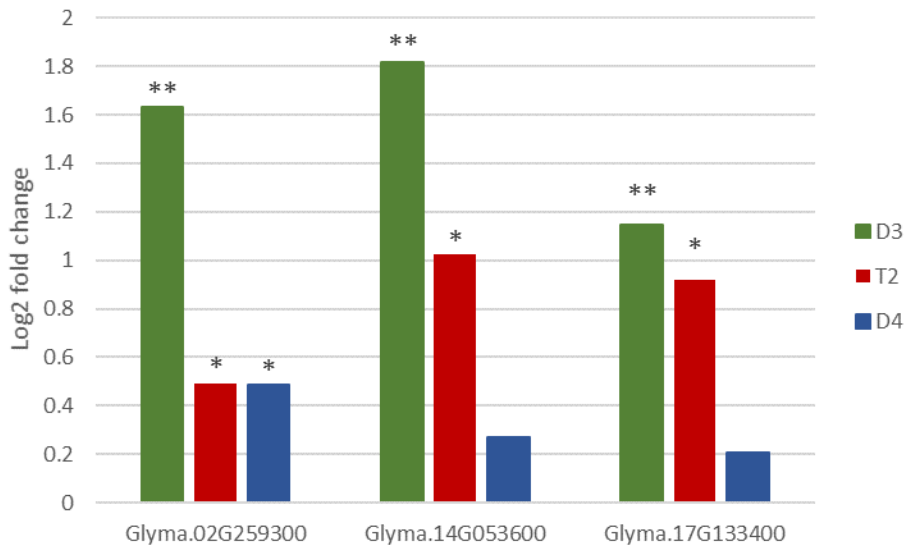

Supplement: Supplementary file 1 [file genes-08-00357-s001.zip › Supplemental_Fig_S2.pdf]

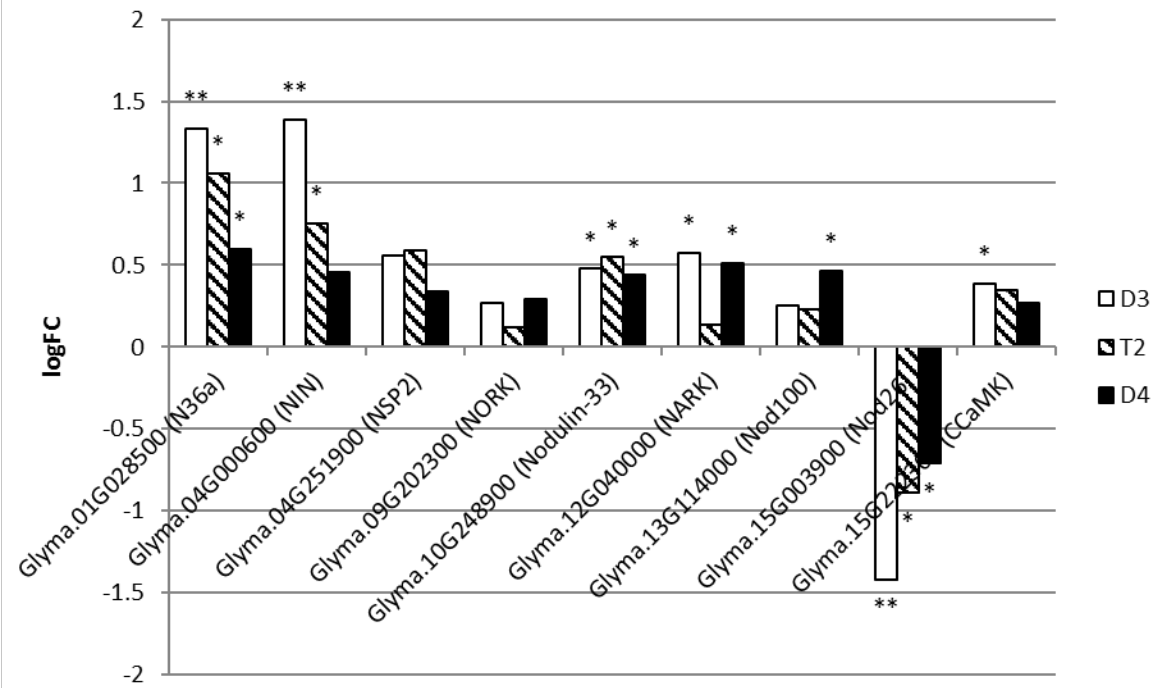

Supplement: Supplementary file 1 [file genes-08-00357-s001.zip › Supplemental_Fig_S3.pdf]

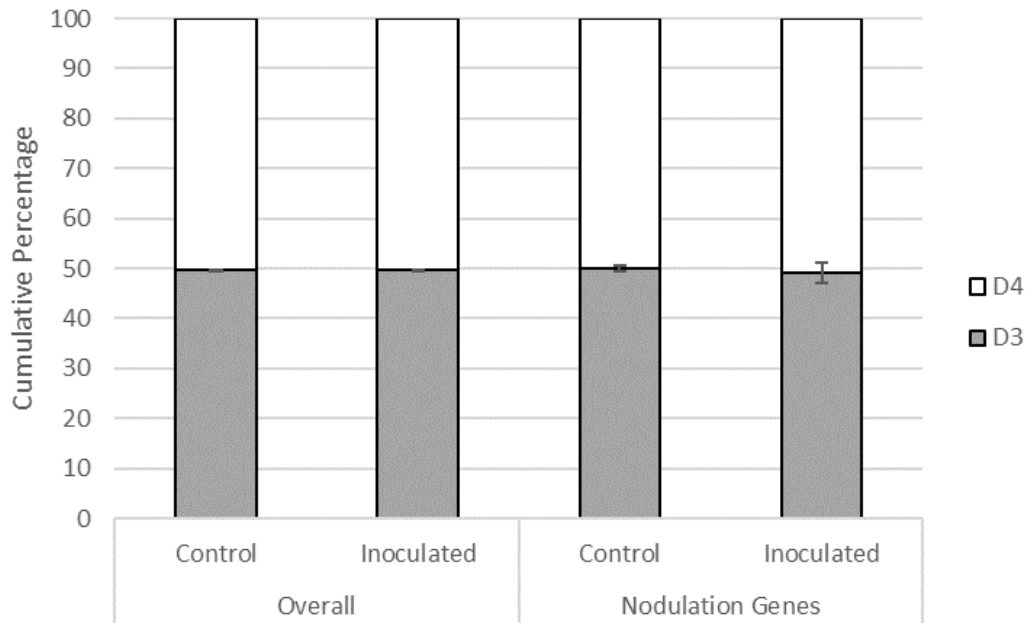

Supplement: Supplementary file 1 [file genes-08-00357-s001.zip › Supplemental_Fig_S4.pdf]

Block 1

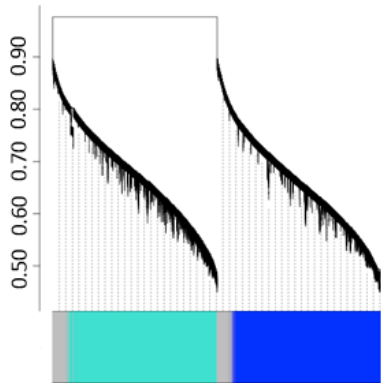

Block 2

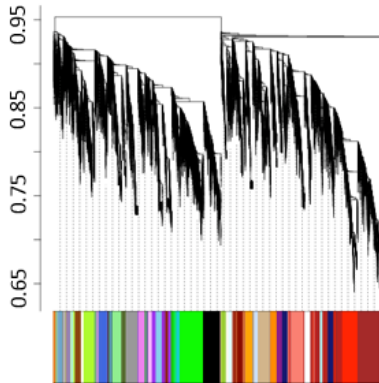

Block 3

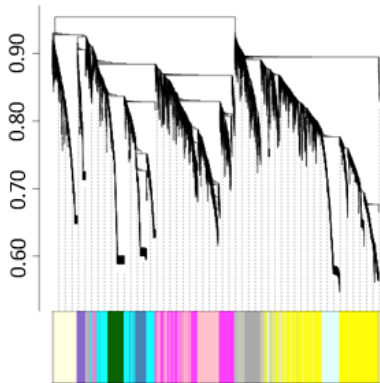

Supplement: Supplementary file 1 [file genes-08-00357-s001.zip › Supplemental_Fig_S5.pdf]

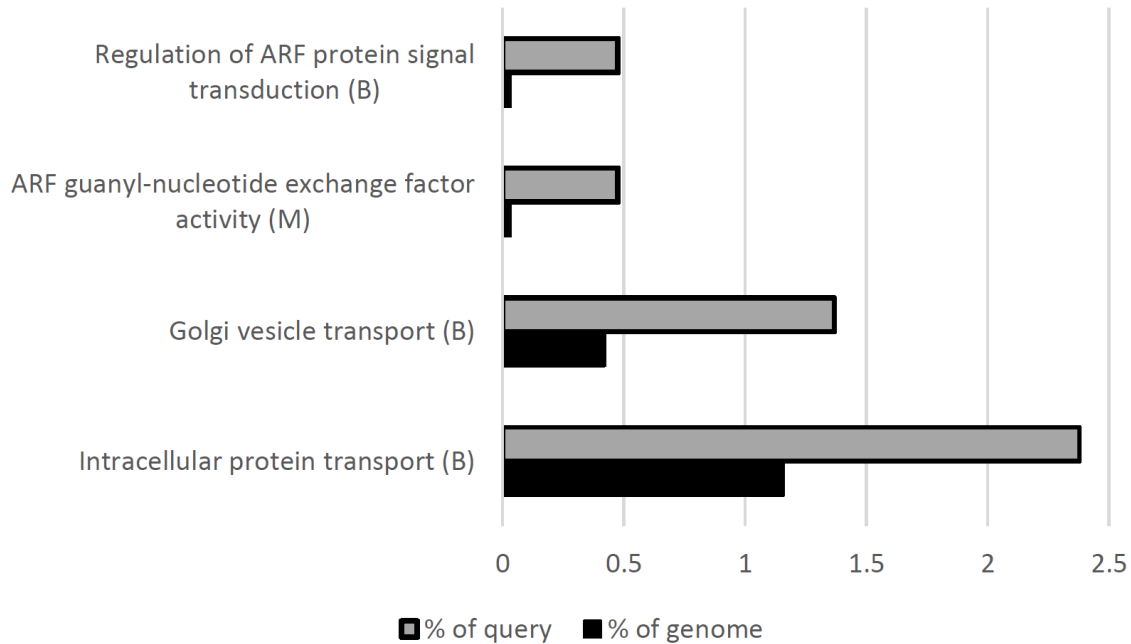

Supplement: Supplementary file 1 [file genes-08-00357-s001.zip › Supplemental_Fig_S6.pdf]

**cor=0.5, p=1.6e-113**

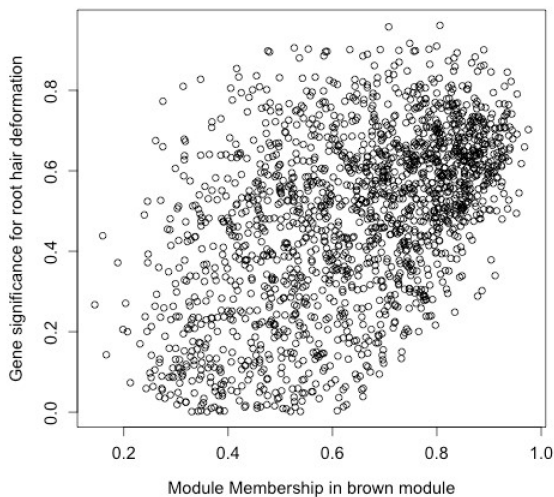

**cor=0.33, p=1.2e-36**

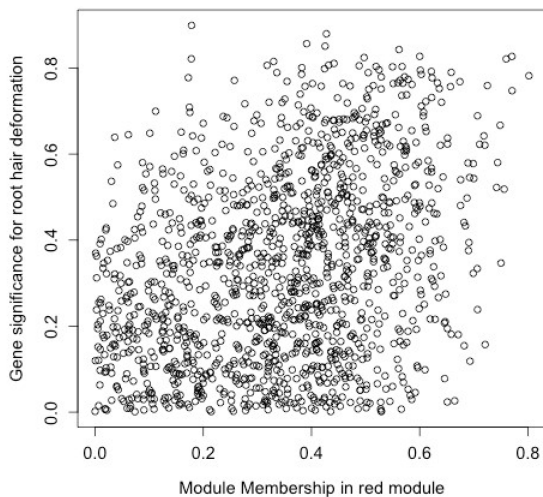

**cor=0.26, p=3.7e-17**

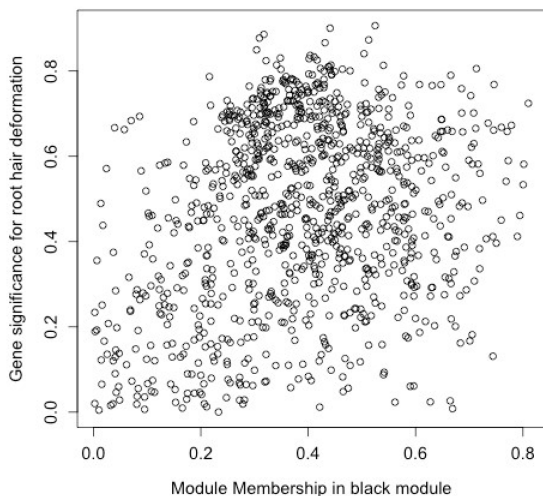

**cor=0.36, p=9.3e-48**

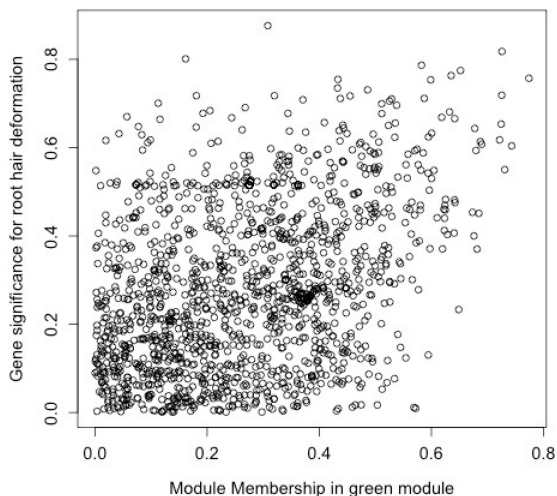

**cor=-0.056, p=0.39**

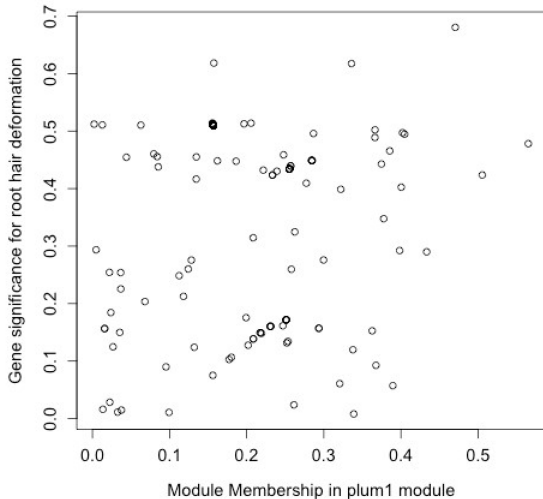

**cor=0.086, p=0.045**

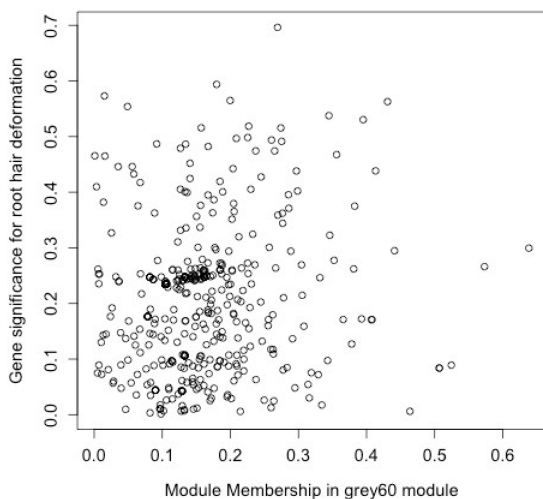

Supplement: Supplementary file 1 [file genes-08-00357-s001.zip › Supplemental_Fig_S7.pdf]
